# Intelligent Sensors for Smart and Autonomous Vehicles

**DOI:** 10.3390/s25216584

**Published:** 2025-10-26

**Authors:** István Barabás, Calin Iclodean, Máté Zöldy

**Affiliations:** 1 Department of Automotive Engineering and Transports, Technical University of Cluj-Napoca, 400001 Cluj-Napoca, Romania; istvan.barabas@auto.utcluj.ro; 2 Department of Automotive Technologies, BME Faculty of Transportation Engineering and Vehicle Engineering, 1111 Budapest, Hungary; zoldy.mate@kjk.bme.hu

## 1. Introduction

Autonomous vehicles (AVs) must exhibit a high degree of intelligence; beyond the functional algorithms implemented within their autonomous driving systems, AVs are also required to independently determine appropriate actions in real time, including in safety-critical scenarios. Achieving higher levels of driving automation—specifically Levels 4 and 5 as defined by the SAE J3016 standard [1]—necessitates the integration of advanced intelligent perception systems [2,3].

Core functions such as localization, environmental perception, behavior prediction, and motion planning rely heavily on data acquired through intelligent sensors embedded within a vehicle. These sensors interpret dynamic environmental inputs and enable informed decision-making by the AV [4].

A comprehensive and up-to-date overview of the capabilities, limitations, advantages, and disadvantages of intelligent sensor technologies is essential for researchers, engineers, and stakeholders engaged in the development of autonomous vehicles. Such knowledge facilitates the creation of AV systems that are not only more reliable and robust but also significantly safer [5].

This Special Issue, entitled “Intelligent Sensors for Smart and Autonomous Vehicles”, addresses the crucial role of advanced sensorial systems in the development of autonomous vehicles (AVs). As AV technologies progress toward higher levels of automation, the need for robust, accurate, and intelligent perception becomes increasingly important. Sensor systems such as RADAR, LiDAR, cameras, ultrasonic devices, GPS/GNSS modules, and Vehicle-to-Vehicle (V2V) communication units form the foundation of environmental awareness in autonomous systems [6].

The integration and fusion of data obtained from these sensors are essential for achieving reliable localization, object detection, classification, and decision-making processes. Moreover, the combination of artificial intelligence algorithms with sensorial inputs enhances a vehicle’s ability to perceive, understand, and respond to complex driving scenarios [7]. Emerging paradigms such as edge and fog computing, alongside the evolution of intelligent proprioceptive and exteroceptive sensors, are shaping the architecture of next-generation AV systems [8].

In this Special Issue, recent research in the field of smart sensors is published, with a focus on increasing the performance and capabilities of these sensors, reducing expenses and constructive dimensions, advancing sensor signal fusion, and decreasing signal transmission time and processing latencies. Advances in edge artificial intelligence and sensor-specific processing enable real-time results while adhering to safety standards [9,10]. Advances in smart sensor technology have converted vehicle sensor systems from static to adaptive sensor networks based on machine learning and which are capable of complex environmental interpretation [11].

Despite significant progress in the field of smart sensors, many knowledge and technology gaps remain, including regarding the robustness of perception in adverse weather conditions and extreme events; reliable calibration and synchronization between heterogeneous sensors; the immaturity of some sensor signal fusion algorithms; insufficient volumes of multimodal datasets; latency in signal transmission, processing, and interpretation; energy efficiency factors; and implementation costs [12]. Furthermore, as connection between smart vehicles and smart environments expands, cybersecurity and data privacy issues become more prevalent [13].

Future research should concentrate on creating verifiable and validatable multimodal fusion systems that allow for real-world uncertainty while maintaining consistent performance under these unpredictable real-world settings [14]. Efforts toward achieving physics-based simulation, long-tail dataset creation, energy-efficient edge inference, and functional safety certification frameworks will be critical in reducing the gap between laboratory innovation and large-scale implementation in intelligent and autonomous vehicles on the road [15,16].

This Special Issue invites the submission of scientific contributions that explore various aspects of intelligent sensing, including conceptual and architectural models, comparisons between real and virtual systems, advanced sensor fusion techniques, cybersecurity in sensor-based systems, and the integration of intelligent sensors into V2X communication networks. By consolidating cutting-edge research in these domains, this Issue aims to support the development of autonomous vehicles that are not only more capable and efficient but also significantly safer and more trustworthy in real-world conditions.

## 2. Short Presentation of the Papers

In the review titled “An Approach to Modeling and Developing Virtual Sensors Used in the Simulation of Autonomous Vehicles” (contribution 1), Barabás et al. present a virtual modeling approach that facilitates the study of real-world phenomena within a simulated environment. This approach is based on a theoretical model that digitally replicates a real system, enabling the analysis and validation of vehicle behavior under various operating conditions. The complexity of the virtual model must reflect the complexity of the actual system under evaluation—being as intricate as necessary, yet as simplified as possible—to ensure the reliability of computer simulations and their validation against experimental measurements.

The virtual model of the autonomous vehicle was developed using CarMaker, version 12.0, a simulation platform created by IPG Automotive. This software is widely adopted both in the international academic community and in the automotive industry, and supports real-time simulation of elementary vehicle systems at the system level and offers an open framework for designing virtual test scenarios in domains such as autonomous driving, advanced driver assistance systems (ADASs), powertrain development, and vehicle dynamics.

This model incorporates a variety of virtual sensors, including slip angle, inertial, object detection, free-space, traffic sign recognition, lane detection, road surface, object-by-line, camera-based, and Global Navigation Satellite System (GNSS) sensors, as well as radar, LiDAR, and ultrasonic sensors. These virtual sensors can be categorized according to their response generation mechanisms: sensors based on parameters derived from measurement characteristics, sensors developed through modeling techniques, and application-specific sensors.

Medina et al., in the review “An Overview of Autonomous Parking Systems: Strategies, Challenges, and Future Directions” (contribution 2), discuss the rapid evolution of Autonomous Parking Systems (APSs), which promise significant improvements in convenience, safety, and operational efficiency. This paper critically examines current strategies in perception, path planning, and vehicle control, as well as system-level considerations such as integration, validation, and security.

Despite notable advancements driven by deep learning techniques and sophisticated sensor fusion, substantial challenges remain. The authors explore the inherent trade-offs, including balancing computational complexity with real-time performance requirements, unresolved foundational issues such as the verification of non-deterministic AI components, and the considerable difficulty of ensuring robust deployment in real-world environments characterized by diverse and unpredictable conditions—ranging from cluttered urban canyons to poorly lit and ambiguously marked parking facilities.

The review further addresses the limitations of existing technologies; the complexities involved in safety assurance within dynamic settings; the pervasive influence of cost constraints on system capabilities; and the critical, often underestimated, importance of establishing genuine user trust. The authors conclude that future research must not only develop innovative technological solutions to these challenges but also engage with the complex socio-technical dimensions necessary to fully realize the potential of Autonomous Parking Systems.

In their review titled “Recent Developments on Drivable Area Estimation: A Survey and a Functional Analysis” (contribution 3), Hortelano et al. highlight that the most advanced Autonomous Driving Systems (ADSs) currently depend heavily on the prior generation of High-Definition (HD) maps. While effective, this process is costly and requires frequent updates to reflect the dynamic nature of road environments. As an alternative, the online generation of accurate navigation maps presents a promising solution to reduce these costs and to extend the Operational Design Domains (ODDs) of modern ADSs.

The authors provide a comprehensive overview of the state of the art in drivable area estimation—an essential component for enabling ADS functionality in environments where HD maps are limited or unavailable. The study proposes a novel architectural framework that encompasses both learning- and non-learning-based methods, facilitating a systematic analysis of recent and impactful algorithms in this field.

Furthermore, the review includes practical resources for researchers and practitioners, such as an evaluation of the impact of modern sensing technologies on drivable area estimation, and a curated list of benchmark datasets relevant for performance evaluation and algorithm comparison.

Tollner et al., in their paper “How Do We Calibrate a Battery Electric Vehicle Model Based on Controller Area Network Bus Data?” (contribution 4), argue that transforming a modern vehicle into a measurement system is a highly beneficial approach, given the wide array of sensors integrated within onboard control and diagnostic systems. As these procedures are not executed by a single control unit, it is essential to transmit signal values over a communication network, enabling an external device to be connected to achieve real-world data acquisition during driving scenarios.

The primary objective of the paper is to utilize the recorded data to validate a one-Degree-Of-Freedom (1-DOF) longitudinal vehicle and powertrain model. To ensure repeatability, three urban driving routes are selected: a flat road, a road with a slight incline, and a road with a steeper gradient, each in both directions. This setup allows the drivetrain system to be tested across a wide load spectrum, including periods of extended energy recuperation. Altitude variations are captured using a Differential GPS (DGPS) system.

Based on the collected data, key vehicle and drivetrain model parameters can be calibrated, including aerodynamic drag coefficients, rolling resistance values, and drivetrain efficiency metrics. Model validation is performed by evaluating speed tracking performance and ensuring that the relative deviation in cumulative energy remains below a 10% threshold.

Ultimately, the developed model is suitable for further energy analysis or the design of control strategies. The study also presents the energy balance of the test cycles as supporting evidence for the model’s accuracy.

Cap et al., in the paper “Hybrid MambaVision and Transformer-Based Architecture for 3D Lane Detection” (contribution 5), demonstrate that lane detection is a critical task in the fields of computer vision and autonomous driving. This includes identifying road markings on the road surface, not only assisting drivers in keeping their vehicles in the proper lane, but also offering essential data for advanced driver assistance systems and autonomous vehicles.

The authors provide a new framework built on the MambaVision-S-1K backbone that combines Mamba-based processing with Transformer capabilities to extract both local detail and global context from monocular pictures. This hybrid approach enables precise three-dimensional simulation of lane geometry, even when elevation varies. By replacing the typical convolutional neural network backbone with MambaVision, the proposed model dramatically enhances 3D lane detection systems. This technique achieves cutting-edge performance on the ONCE-3DLanes dataset, demonstrating its supremacy in precisely capturing lane curvature and elevation changes. These findings demonstrate the possibility of integrating enhanced backbones based on Vision Transformers into autonomous driving systems to improve lane detection robustness and reliability.

Radu et al., in their paper “Design and Validation of an Active Headrest System with Integrated Sensing in Rear-End Crash Scenarios” (contribution 6), address the significant safety challenges posed by rear-end collisions, particularly due to the high risk of whiplash injuries among vehicle occupants. The accurate simulation of occupant kinematics during such impacts is essential for the development of advanced occupant protection systems.

This paper introduces an enhanced multibody simulation model tailored specifically for rear-end crash scenarios, integrating active headrest mechanisms and sensor-based activation logic. The model features detailed representations of vehicle structural components, suspension systems, restraint systems, and occupant biomechanics, enabling precise prediction of crash dynamics and occupant responses.

The system was developed using Simscape Multibody, with components derived from CAD models and interconnected through physically realistic joints. Validation was conducted through controlled experimental crash tests, with particular emphasis placed on accurately modeling contact forces, suspension dynamics, and actuator response times within the active headrest system.

The model demonstrated high predictive accuracy, achieving a root mean square error (RMSE) of 4.19 m/s^2^ and a Mean Absolute Percentage Error (MAPE) of 0.71% in head acceleration measurements during frontal impact tests, confirming the model’s reliability in replicating occupant kinematics and head acceleration profiles.

This research underscores the importance of integrated sensor–actuator systems in enhancing occupant safety and establishes a robust, adaptable platform for future investigations into intelligent vehicle safety technologies.

Beles et al., in their paper “Driver Drowsiness Multi-Method Detection for Vehicles with Autonomous Driving Functions” (contribution 7), present a comprehensive approach to the development of a fuzzy-logic-based decision algorithm aimed at detecting and issuing warnings regarding driver drowsiness. The proposed algorithm integrates an analysis of ElectroOculoGraphy (EOG) signals and eye state images to assess the driver’s level of alertness, with the primary goal of preventing accidents caused by reduced attentiveness.

The drowsiness detection system consists of several core components responsible for acquiring data, analyzing physiological and visual indicators, and making decisions regarding the driver’s state of vigilance. When signs of drowsiness are detected, the system can issue appropriate warnings to alert the driver.

Driver drowsiness is typically manifested through a progressive decline in attention to road and traffic conditions, impaired driving performance, and increased reaction times—all of which significantly elevate the risk of accidents. In scenarios where the driver fails to respond to repeated alerts, the advanced driver assistance system (ADAS) is expected to intervene by taking control of the vehicle to ensure continued safety.

This research highlights the critical role of multi-method detection systems and fuzzy logic decision-making in enhancing safety in semi-autonomous and autonomous driving environments.

Montiel-Marín et al., in their paper “Point Cloud Painting for 3D Object Detection with Camera and Automotive 3+1D RADAR Fusion” (contribution 8), discuss the longstanding integration of RADAR and camera sensors in automotive systems since the inception of advanced driver assistance systems (ADASs). While these sensors exhibit complementary strengths and weaknesses, their potential has been relatively underexplored within learning-based object detection methods.

This work proposes a novel approach for object detection in autonomous driving by employing a geometrical and sequential sensor fusion technique that combines 3+1D RADAR data with semantic information extracted from camera imagery via point cloud painting from the perspective view. To this end, the authors adapt the PointPainting method—originally developed for LiDAR and camera data—to the fusion of 3+1D RADAR and camera sensors.

The process begins with the application of YOLOv8-seg to generate instance segmentation masks, which are subsequently projected onto the RADAR point cloud. A set of heuristic rules is introduced as a refinement step to reduce error propagation from the segmentation phase to the detection phase. The pipeline concludes with the application of PointPillars as the object detection network operating on the painted RADAR point cloud.

The proposed approach is validated using the novel View of Delft dataset, which contains 3+1D RADAR sequences captured in urban environments. Experimental results demonstrate that the fusion of RADAR and camera data significantly outperforms the RADAR-only baseline, achieving an increase in mean Average Precision (mAP) from 41.18 to 52.67, corresponding to a relative improvement of 27.9%.

Zhao et al., in their study “YOLOv7-TS: A Traffic Sign Detection Model Based on Sub-Pixel Convolution and Feature Fusion” (contribution 9), report significant advancements in deep learning-based object detection in recent years. Within this domain, traffic sign detection represents a critical subtask with substantial potential for development; however, existing object detection methods for traffic signs in real-world scenarios often suffer from challenges such as missed detections of small objects and low overall detection accuracy.

To address these limitations, the authors propose a novel traffic sign detection model named YOLOv7-Traffic Sign (YOLOv7-TS), which leverages sub-pixel convolution and features fusion techniques. Firstly, the model exploits the up-sampling capability of sub-pixel convolution by integrating the channel dimension and introduces a Feature Map Extraction Module (FMEM) to reduce channel information loss. Furthermore, a Multi-feature Interactive Fusion Network (MIFNet) is designed to enhance information interaction across all feature layers, thereby improving feature fusion effectiveness and augmenting the capability of detecting small objects. Additionally, a Deep Feature Enhancement Module (DFEM) is incorporated to accelerate the pooling process while enriching features at the highest layer.

YOLOv7-TS is evaluated on two traffic sign datasets: CCTSDB2021 and TT100K. Compared to the baseline YOLOv7 model, YOLOv7-TS achieves notable improvements in mean Average Precision (mAP) of 3.63% and 2.68% on the respective datasets, while employing a smaller number of parameters. These results demonstrate the effectiveness of the proposed model.

Gong et al., in their paper “Identification of Driver Status Hazard Level and the System” (contribution 10), highlight that according to survey statistics, the majority of traffic accidents result from irregularities in driver behavior and status. Due to the absence of a multi-level hazardous state grading system both domestically and internationally, this paper proposes a comprehensive state grading system for real-time detection and dynamic tracking of driver status.

The system employs an OpenMV acquisition camera combined with a cradle head tracking mechanism to dynamically capture real-time images of the driver’s current driving behavior. It integrates the YOLOX with the OpenPose algorithm to identify dangerous driving behaviors by detecting unsafe objects within the cabin and analyzing the driver’s posture. Additionally, an improved RetinaFace face detection algorithm is combined with the Dlib feature-point algorithm to assess driver fatigue.

Experimental results demonstrate that the proposed system achieves accuracies of 95.8%, 94.5%, and 96.3% in detecting three defined driver hazard levels (R1, R2, and R3), respectively. These findings indicate that the system holds practical significance for real-time warnings against driver distraction and related unsafe behaviors.

Ghang et al., in their paper “Cyclist Orientation Estimation Using LiDAR Data” (contribution 11), emphasize the importance of predicting cyclist behavior in autonomous vehicle decision-making. When navigating real traffic environments, a cyclist’s body orientation indicates their current direction of movement, while head orientation reflects their intention to observe the road situation before making subsequent maneuvers. Consequently, accurate estimation of both body and head orientations is a critical component of cyclist behavioral prediction in autonomous driving.

This research proposes two deep-neural-network-based methods for estimating cyclist orientation—encompassing both body and head—using data from Light Detection and Ranging (LiDAR) sensors. The first method represents LiDAR data through 2D images encoding reflectivity, ambience, and range information. The second method directly utilizes 3D point cloud data to represent the sensor’s output. Both approaches employ ResNet50, a 50-layer convolutional neural network, for orientation classification.

The study compares the performance of these two methods to determine the most effective use of LiDAR sensor data for cyclist orientation estimation. A dedicated cyclist dataset was developed, comprising multiple cyclists exhibiting diverse body and head orientations.

Experimental results indicate that the model based on 3D point cloud data outperforms the 2D-image-based model in cyclist orientation estimation. Furthermore, within the 3D point cloud approach, the use of reflectivity information yields more accurate estimations than ambient information.

Kibii et al., in their paper “Design and Calibration of Plane Mirror Setups for Mobile Robots with a 2D-Lidar” (contribution 12), discuss the widespread use of LiDAR sensors for environmental perception in Autonomous Robot Vehicles (ARVs). They note that the Field Of View (FOV) of LiDAR sensors can be effectively reshaped by strategically positioning plane mirrors in their vicinity. Such mirror setups are particularly advantageous for enhancing ground detection capabilities in ARVs equipped with 2D LiDAR sensors.

This paper presents an overview of several geometric mirror designs and evaluates their strengths for various vehicle types. Furthermore, the authors introduce a novel, easy-to-implement calibration procedure for 2D LiDAR mirror setups, aimed at precisely determining mirror orientations and positions. This method utilizes a single flat calibration object featuring a pre-aligned, simple fiducial marker.

The study presents measurement data collected from a prototype vehicle equipped with a 2D LiDAR sensor (with a 2 m range) employing this calibration procedure. The results demonstrate that the calibrated mirror orientations achieve an accuracy of less than 0.6° within this short range, representing a significant improvement over orientation angles derived directly from CAD models. The accuracy of the resulting point cloud data improves without any notable increase in distance noise.

Based on these findings, the authors propose general guidelines for successful calibration using their method. In conclusion, a 2D LiDAR sensor combined with two plane mirrors calibrated via this approach offers a cost-effective and precise solution for robot engineers seeking to enhance the environmental perception capabilities of ARVs.

Peiris et al., in their study “Quantifying the Foregone Benefits of Intelligent Speed Assist Due to the Limited Availability of Speed Signs across Three Australian States” (contribution 13), demonstrate that Intelligent Speed Assist (ISA), which communicates speed limits to drivers via speed sign recognition cameras, is expected to yield significant road safety improvements by enhancing speed compliance. However, due to the absence of comprehensive digital speed maps and limited cellular connectivity across Australia, this study estimates the potential safety benefits foregone when ISA relies solely on physical speed signs for optimal advisory function.

The analysis begins by identifying speed-related Fatalities and Serious Injuries (FSIs) in the Australian states of Victoria, South Australia, and Queensland over the period 2013–2018. Published ISA effectiveness estimates are then applied to quantify the potential safety benefits of ISA deployment. Taking into account the presence and absence of speed signage across these states, the study estimates the foregone savings as the proportion of FSIs that would remain unprevented due to missing speed sign infrastructure.

The results indicate that annually, 27–35% of speed-related FSIs in each state are unlikely to be prevented by ISA because of inadequate speed sign coverage, corresponding to economic losses estimated at between AUD 62 million and AUD 153 million. Despite several assumptions regarding ISA fitment rates and driver acceptance, conservative estimates suggest that the consistent placement of speed signs across various road classes and remoteness levels would substantially outweigh the costs associated with current speed sign deficiencies.

This study introduces a novel methodology for quantifying the foregone benefits of ISA attributable to suboptimal road infrastructure. It provides a valuable framework for prioritizing infrastructure investments to maximize the safety gains achievable through advanced driver assistance technologies.
